# The complete mitochondrial genome of *Vibrissina turrita* (Meigen, 1824) (Diptera, Tachinidae)

**DOI:** 10.1080/23802359.2024.2363344

**Published:** 2024-06-17

**Authors:** Hui Yang, Chuntian Zhang, Yahui Zhang, Junjian Li

**Affiliations:** College of Life Sciences, Shenyang Normal University, Shenyang, China

**Keywords:** Mitogenome, phylogeny, Exoristinae

## Abstract

In this study, the mitogenome of *Vibrissina turrita* (Meigen, 1824) (Diptera, Tachinidae) was sequenced based on the next-generation sequencing approach and analyzed here for the first time. The 17,387 bp genome has a high A + T content and consists of 13 protein-coding genes, 22 transfer RNA genes, two ribosomal RNA genes, and one noncoding control region. The phylogenetic analysis results support that Exoristinae is monophyletic and *V. turrita* belongs to the subfamily. This study reveals the systematic classification status of *V. turrita* and will enrich the genetic data on Tachinidae.

## Introduction

Exoristinae is the largest subfamily in the family Tachinidae (Diptera), which has 3,687 described species worldwide (O’Hara et al. 2020). As a parasitoid, it is a significant natural enemy in natural and managed terrestrial ecosystems, particularly in forests (Yan et al. 2021). It has a wide range of hosts, mainly parasitizing on larvae of Lepidoptera, and is also a natural enemy of certain Orthoptera, Hymenoptera, and other insect orders (Stireman et al. 2006). *Vibrissina* Rondani belongs to the Blondeliini, with a total of 43 species, of which three have been recorded in China (O’Hara et al. 2020). Mitochondrial genome has been widely used in phylogenetic studies or molecular level species identification of Diptera insects, e.g. Yan et al. (2021) and Zhou et al. (2024). But only a few complete mitochondrial genomes of the Tachinidae have been published. In this study, we sequenced the mitogenome data of *V. turrita* (Meigen, 1824) and annotated it as the first mitogenome of genus *Vibrissina*, which will contribute to gaining an understanding of the phylogenetic relationships of *V. turrita* and future genetic research in the family.

## Materials and methods

**Figure 1.**
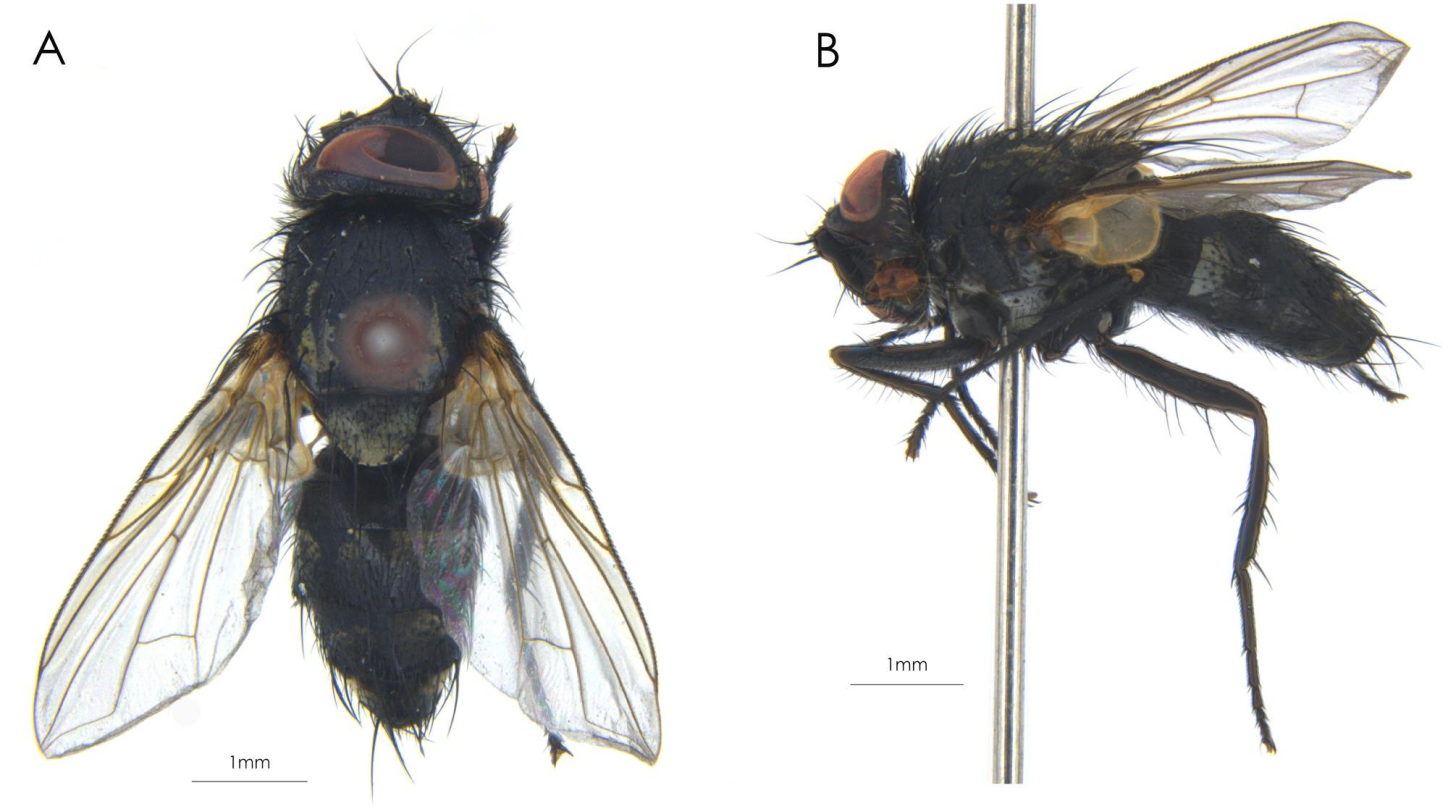
A female adult of *Vibrissina turrita* (Meigen, 1824). (A) Dorsal view; (B) lateral view. Scale bars: 1.0 mm. The photos were taken by Yahui Zhang (under the voucher number SYNU20210815).

The adult specimens of *V. turrita* (Figure 1) were collected by net-trap method from Milin City, Linzhi City of Xizang Autonomous Region (=Tibet), China (29.3155° N, 94.4566° E) by Junjian Li on 8 August 2021, and are deposited at the Insects Collection of Shenyang Normal University (Junjian Li, ljj881014@163.com). Species identification follows Cumming and Wood (2017) and Tschorsnig and Richter (1998): eyes bare. Frons about 2/3 (male) or 3/4 (female) of eye width. Parafacial bare. Facial ridge with usually strong setae on lower 1/3 to 4/5. Occiput mostly has white hairs or setulae. Postpedicel 6–8 times as long as pedicel; arista bare. Prosternum hairy; three presutural and three postsutural dorsocentral setae; 1st postsutural supra-alar seta shorter than notopleural setae and 1st postsutural intra-alar seta; apical scutellar setae absent; proepisternum bare; three postpronotal setae not arranged in a triangle; two katepisternal setae. Mid tibia with one anterodorsal and one ventral seta. Abdominal syntergite 1 + 2 is not medially excavated to posterior margin, tergites 3 and 4 have median discal setae. Female with piercer, ventral margins of tergites 3 and 4 usually with spinules. The specimens were examined with Zeiss Stemi SV11 stereomicroscopes and stored at −20 °C for subsequent DNA extraction.

According to the manufacturer’s protocol, a QIAamp Micro DNA Kit (Qiagen, Hilden, Germany) was used to extract DNA from the female adult’s muscle tissues of the thorax (the voucher number for the sample is SYNU20210808). Genomic DNA was randomly fragmented by Covaris (Covaris, LLC, Woburn, MA), followed by fragments selection by Agencourt AMPure XP-Medium Kit (Beckman, Brea, CA) to an average size of 200–400 bp. Selected fragments were end repaired and 3′ adenylated, then the adaptors were ligated to the ends of these 3′ adenylated fragments. Finally, the single strand circle DNA was formatted as the final library. DNBSEQ-T7 (MGI, Shenzhen, China) was used to perform paired-end sequencing with a read length of 150 bp (PE 150 bp).

**Figure 2.**
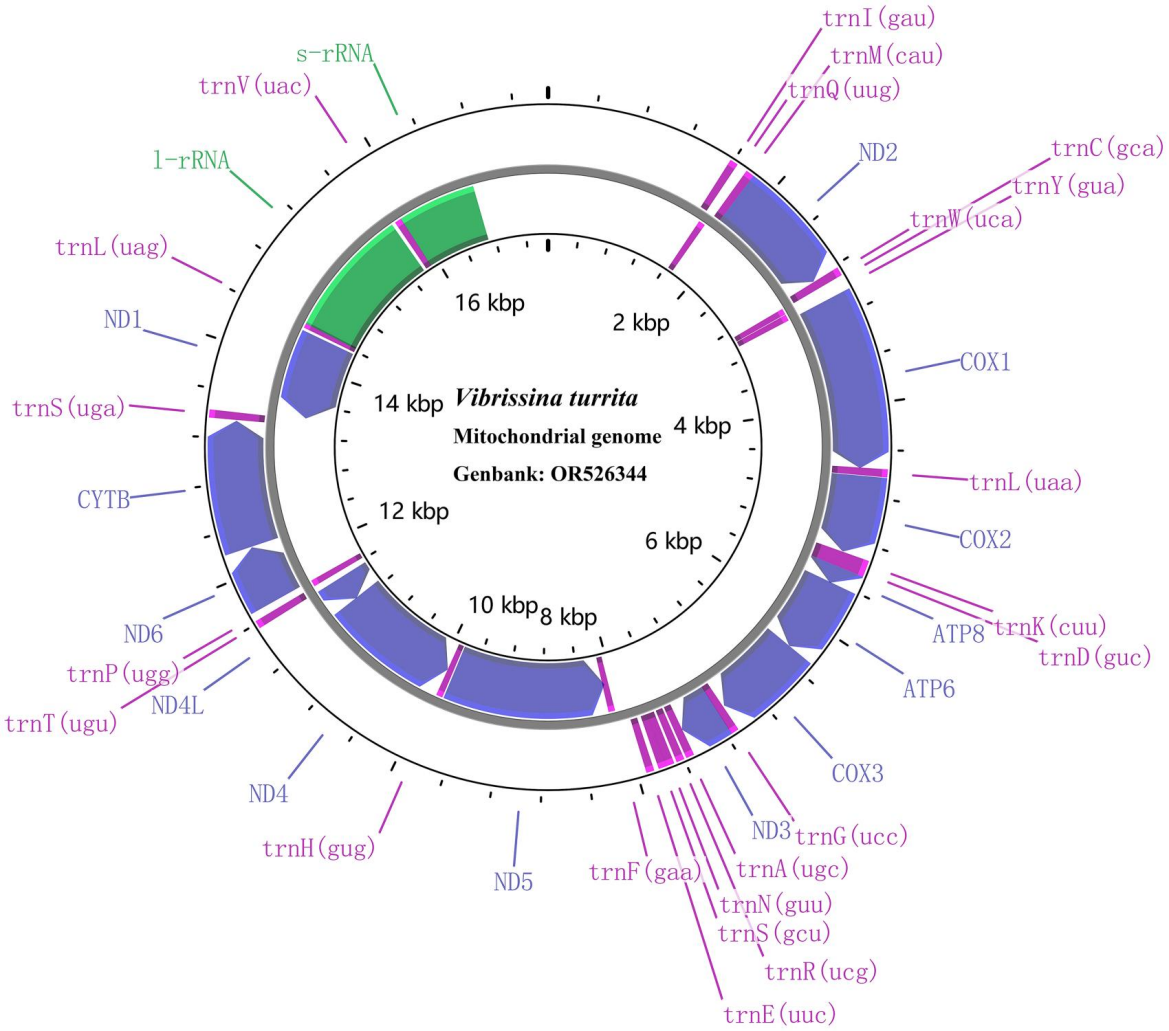
Circular gene map of the *V. turrita* mitochondrial genome. The genes displayed inside and outside the circle are transcribed counterclockwise and clockwise, respectively. Purple represents tRNA genes, blue represents protein-coding genes, and green represents rRNA genes.

The raw data were assembled using Mitoz 3.6 (Meng et al. 2019). We compared the sequences of the Tachinidae and manually annotated the sequence of *V. turrita*, and then the data were submitted to GenBank database through NCBI. The E-INSi method of MAFFT (Katoh and Standley 2013) was used to align the 13 protein-coding genes (PCGs) and then trim them by trimAL (Capella-Gutiérrez et al. 2009). Circular maps of the mitogenomes were prepared using Proksee (Grant et al. 2023) (Figure 2).

To investigate the phylogenetic status of *V. turrita*, we used the mitogenome sequences of 23 species in the family Tachinidae, which included 17 sequences available for all subfamily Exoristinae in the GenBank database when we conducted this study. The mitogenome sequences of *Lucilia sericata* (Calliphoridae) and *Sarcophaga crassipalpis* (Sarcophagidae) were used as outgroups. The maximum-likelihood (ML) reconstruction was performed using IQ-TREE (Nguyen et al. 2015) with bootstrap set to 5000. The alignment, trimming, data concatenation, and tree building of sequences are all completed in PhyloSuite 1.2.2 (Zhang et al. 2020).

## Results

The complete mitogenome of *V. turrita* (GenBank accession number: OR526344) is 17,387 bp in length. A total of 37 genes were annotated, consisting of 13 PCGs, two rRNA genes, 22 tRNA genes, and one non-coding region. The coverage depth map of *V. turrita* is detailed in Supplementary Figure S1. The nucleotide composition was 42.01% of A, 39.13% of T, 11.26% of C, 7.60% of G, and 81.14% of A + T content. Most of the 13 PCGs used ATN as the start codon (ATG for *CYTB*, *ND4*, *ND4L*, *COX2*, *COX3*, and *ATP6*; ATT for *ND1*, *ND2*, *ND5*, and *ND6*; ATA for *ND3* and ATC for *ATP8*), except that *COX1* begins with codon TCG. The stop codon TAA is used to most of the PCGs (*ND2*, *COX1*, *COX3*, *ATP8*, *ATP6*, *ND4L*, *CYTB*, and *ND6*), whereas incomplete stop codon T is used by three PCGs (*ND4*, *ND5*, and *COX2*); *ND3* and *ND1* terminate with the codon TAG.

**Figure 3.**
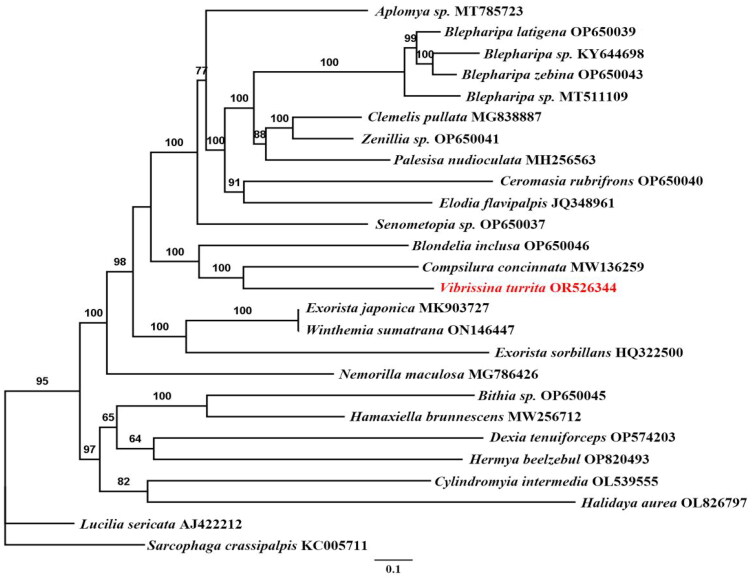
Phylogenetic tree of 23 tachinid species based on the concatenated dataset of 13 PCGs using the maximum-likelihood (ML) method. Nodal support values indicate the maximum-likelihood bootstrap support value (BP). Accession numbers of mitochondrial sequences used in the phylogenetic analysis are listed after scientific name. The focal species is highlighted in red font. The nucleotide sequences of the 13 PCGs of following species were also used: *Aplomya* sp. MT785723 (unpublished), *Blepharipa latigena* OP650039 (unpublished), *Blepharipa* sp. KY644698 (unpublished), *Blepharipa zebina* OP650043 (unpublished), *Blepharipa* sp. MT511109 (unpublished), *Clemelis pullata* MG838887 (unpublished), *Zenillia* sp. OP650041 (unpublished), *Palesisa nudioculata* MH256563 (unpublished), *Ceromasia rubrifrons* OP650040 (unpublished), *Elodia flavipalpis* JQ348961 (Zhao et al. 2013), *Senometopia* sp. OP650037 (unpublished), *Blondelia inclusa* OP650046 (unpublished), *Compsilura concinnata* MW136259 (Luo et al. 2021), *Exorista japonica* MK903727 (Seo et al. 2019), *Winthemia sumatrana* ON146447 (unpublished), *Exorista sorbillans* HQ322500 (Shao et al. 2012), *Nemorilla maculosa* MG786426 (unpublished), *Bithia* sp. OP650045 (unpublished), *Hamaxiella brunnescens* MW256712 (unpublished), *Dexia tenuiforceps* OP574203 (unpublished), *Hermya beelzebul* OP820493 (unpublished), *Cylindromyia intermedia* OL539555 (unpublished), *Halidaya aurea* OL826797 (unpublished), *Lucilia sericata* AJ422212 (Palevich et al. 2021), and *Sarcophaga crassipalpis* KC005711 (Ramakodi et al. 2015).

The results of the phylogenetic tree (Figure 3) show that the *V. turrita* belongs to the Exoristinae. The relationship between the genus *Vibrissina* and *Compsilura* is relatively close in the phylogenetic tree with 100% bootstrap support. The complete mitogenome of *V. turrita* will contribute to the in-depth research on molecular bases for the taxonomic system and phylogeny of Tachinidae.

## Discussion and conclusions

The mitogenome sequences of *V. turrita* are similar in gene arrangements, gene number, and nucleotide composition with the other species in the Blondeliini, as a typical mitogenome. Because of the variation of length in the A + T rich region, the length of mitochondrial genome is longer than the other reference species in the same tribe.

The phylogenetic results show that a relatively close relationship between *Vibrissina* and *Compsilura*, both belonging to the tribe Blondeliini in known classification systems (O’Hara et al. 2020). This result is consistent with the previous work of Stireman et al. (2019). Our works provided the phylogenetic information of Tachinidae at the mitochondrial genome level for inferring the phylogenetic relationship of Diptera species.

## Data Availability

The genome sequence data that support the findings of this study are openly available in GenBank of NCBI at under the accession no.OR526344. The associated BioProject, SRA, and Bio-Sample numbers are PRJNA1043253, SRR27406048, and SAMN38338530, respectively.
